# Impact of Nanoclays on the Biodegradation of Poly(Lactic Acid) Nanocomposites

**DOI:** 10.3390/polym10020202

**Published:** 2018-02-17

**Authors:** Edgar Castro-Aguirre, Rafael Auras, Susan Selke, Maria Rubino, Terence Marsh

**Affiliations:** 1 School of Packaging, Michigan State University, East Lansing, MI 48824, USA; castroag@msu.edu (E.C.-A.); sselke@msu.edu (S.S.); mariar@msu.edu (M.R.); 2 Department of Microbiology and Molecular Genetics, Michigan State University, East Lansing, MI 48824, USA; marsht@msu.edu

**Keywords:** montmorillonite, halloysite, Laponite^®^, composting, biofilm, degradation, bio-based

## Abstract

Poly(lactic acid) (PLA), a well-known biodegradable and compostable polymer, was used in this study as a model system to determine if the addition of nanoclays affects its biodegradation in simulated composting conditions and whether the nanoclays impact the microbial population in a compost environment. Three different nanoclays were studied due to their different surface characteristics but similar chemistry: organo-modified montmorillonite (OMMT), Halloysite nanotubes (HNT), and Laponite^®^ RD (LRD). Additionally, the organo-modifier of MMT, methyl, tallow, bis-2-hydroxyethyl, quaternary ammonium (QAC), was studied. PLA and PLA bio-nanocomposite (BNC) films were produced, characterized, and used for biodegradation evaluation with an in-house built direct measurement respirometer (DMR) following the analysis of evolved CO_2_ approach. A biofilm formation essay and scanning electron microscopy were used to evaluate microbial attachment on the surface of PLA and BNCs. The results obtained from four different biodegradation tests with PLA and its BNCs showed a significantly higher mineralization of the films containing nanoclay in comparison to the pristine PLA during the first three to four weeks of testing, mainly attributed to the reduction in the PLA lag time. The effect of the nanoclays on the initial molecular weight during processing played a crucial role in the evolution of CO_2_. PLA-LRD5 had the greatest microbial attachment on the surface as confirmed by the biofilm test and the SEM micrographs, while PLA-QAC0.4 had the lowest biofilm formation that may be attributed to the inhibitory effect also found during the biodegradation test when the QAC was tested by itself.

## 1. Introduction

Biodegradable polymers like poly(lactic acid) (PLA), poly(butylene adipate-*co*-terephthalate) (PBAT), and thermoplastic starch (TPS), have great potential to replace fossil-based polymers, avoid landfill disposal of most non-recyclable polymers, and help reduce environmental impacts. However, these materials have some properties and processing shortcomings that have limited their use in many applications, for example, brittleness, water sensitivity, low heat distortion temperature, medium to high gas permeability, and low melt viscosity [1,2]. Therefore, the creation of bio-nanocomposites (BNCs) in which the reinforcements have at least one dimension in the nanoscale dimension and the matrix is a biodegradable polymer, preferably a bio-based polymer, has garnered attention [1,3,4]. Ideally, BNCs could be recycled or treated together with other organic wastes in composting facilities and produce compost, a valuable soil conditioner and fertilizer [5].

One particularly useful class of nanofillers used to produce BNCs is inorganic layered silicate minerals, or nanoclays, due to their commercial availability, low cost, significant property enhancement and relatively simple processability [3]. Natural nanoclays, such as montmorillonite (MMT) with chemical structure [Na_0.38_K_0.01_][Si_3.92_Al_0.07_O_8_][Al_1.45_Mg_0.55_O_2_(OH)_2_]·7H_2_O, and synthetic nanoclays, such as Laponite^®^ RD (LRD) with chemical structure Na_0.7_[(Si_8_Mg_5.5_Li_0.3_) O_20_(OH)_4_]_0.7_, and halloysite nanotubes (HNT) with chemical structure Al_2_(OH)_4_Si_2_O_5_(2H_2_O), offer a unique route for enhancing the mechanical, physical and barrier properties of biodegradable polymers at low levels of loading (<5 wt %), especially when the nanoclay particles are well dispersed in the polymer matrix [2,6]. However, the dispersion of hydrophilic nanofillers in a polymer matrix is challenging. Organophilization, or organic modification, is a technique that improves clay compatibility with organic polymers by reducing the surface energy between the clay layers. Increasing the clay inter-gallery spacing facilitates the intercalation and exfoliation of the clay in the polymer matrix [2,3]. The exfoliation into individual layers depends on the clay’s ability for surface modification in which the interlayer inorganic ions are exchanged with organic cations [4,7].

The most broadly studied organo-modifiers are ammonium alkyls. When the clay inorganic ions are exchanged with these organic cations, the inter-gallery spacing increases due to the bulkiness of the alkyl–ammonium ions [7]. For example, organo-modified montmorillonite (OMMT), in which its inorganic ions (e.g., Na^+^, K^+^, Ca^2+^, and Mg^2+^) have been replaced by organic alkyl-ammonium ions, improving the wetting with the polymer chains [1,3]. Several researchers have reported improvement in the properties and performance of PLA with addition of OMMT. For example, Ray et al., through a series of papers, demonstrated that the addition of montmorillonite has a significant effect in the improvement of PLA properties (in both solid and melt states), crystalline behavior, and biodegradability in comparison with pristine PLA. Among the different mechanical properties that have been improved are storage modulus, flexural modulus, flexural strength, tensile modulus and elongation at break [8,9,10]. Additional benefits in performance have been reported such as increased glass transition and thermal degradation temperatures [3,11]. Another reported advantage, other than enhancement of the mechanical and thermal properties, is improvement in the barrier properties due to the enhanced tortuous path provided by the silicate layers to gases like oxygen [9,12,13]. The decreased transparency is a minor disadvantage of these BNCs [3]. Other researchers have found significant improvement in thermo-mechanical and barrier properties of BNCs based on PLA and OMMT [14,15].

Halloysite is another type of nanoclay that has received great attention as filler for polymer/clay nanocomposites due to its biocompatibility, natural abundance, and relatively low cost. HNT has almost no surface charge and does not require organic modification for adequate dispersion [16,17]. However, functionalized HNT has shown improved dispersion during processing and enhanced mechanical and thermal properties [18,19]. HNT has been used as filler for several polymers like poly(propylene) (PP), vinyl ester, polyamide (PA), poly(vinyl chloride) (PVC), epoxy, and natural rubber for enhancing properties such as mechanical, thermal, crystallinity, and fire resistance [18,19]. Researchers have found that PLA-HNT nanocomposites exhibited improvement in properties like tensile strength, Young’s modulus, impact properties, flexural properties, and storage modulus, but no significant modification in the thermal properties in comparison with pure PLA [16,20,21,22]. The addition of HNT promotes crystallization and formation of different crystalline phases [21,22]. HNT was also found to slightly increase water absorption [23]. Other researchers found increased thermal and flame retardant properties besides improvement in mechanical properties [19]. Esma et al. also found enhanced thermal properties, but in their case mechanical properties were not significantly improved [24]. Similarly, Kim et al. found decreased tensile strength with clay loading higher than 5 wt % but enhanced rheological properties [17].

Laponite^®^ (LRD), another clay that might lead to novel properties, has not been widely investigated for the development of PLA-based nanocomposites. LRD is an entirely synthetic hectorite clay that belongs to the group of smectite phyllosilicate minerals, and it has great capacity for swelling and exfoliation [25,26]. The advantage of using synthetic clays like LRD is the high structural regularity, single layer dispersions of nanoparticles, and low level of impurities (e.g., silica, iron oxides, and carbonates). Due to its gelation properties, LRD has been used for different pharmaceutical and cosmetic applications; for example, toothpastes, creams, and glazes [27,28,29,30]. Zhou et al. studied PLA-LRD composite films and found improvement in the thermal stability, tensile strength and hydrophilicity of PLA, especially when the LRD content is below 0.2 wt %. [31,32]. Similarly, Tang et al. studied nanocomposites based on starch, poly vinyl alcohol (PVOH), and LRD and found that an increase in LRD content (0–20%) enhanced tensile strength and decreased water vapor permeability [26].

Besides performance limitations, one of the drawbacks of some biodegradable polymers, like PLA, is that they do not biodegrade as fast as other organic wastes during composting, which in turn affects their general acceptance in industrial composting facilities [33]. Therefore, increasing their biodegradation rate in the composting environment should facilitate and encourage their disposal through these facilities by degrading in a time frame comparable with other organic materials.

Several researchers studied the effect of OMMT on the biodegradation of biodegradable polymers like polycaprolactone (PCL) [34], poly(3-hydroxybutyrate-*co*-3-hydroxyvalerate) (PHBV) [35], TPS [36], and PLA [10,33,37,38,39,40,41,42,43,44,45]. Their results indicated that, in general, these BNCs biodegraded faster than their respective pristine polymer. Therefore, the incorporation of nanoclays into a biodegradable polymer matrix represents a promising approach not only for enhancing the polymer performance but also for increasing its biodegradation rate in composting conditions. However, the effect of different nanoclays and organo-modifiers on the abiotic and biotic degradation of PLA is still unclear and needs further investigation. Even though it is well known that the biodegradation mechanism of PLA involves chemical hydrolysis, the role of microorganisms and how they are affected by the presence of nanoparticles is still not well understood [44].

Thus, this study aimed to understand the biodegradation mechanisms of BNCs made of PLA and compounded with OMMT, HNT, and LRD, and to identify the main factors contributing to their biodegradation rate such as those related to the polymer structure and also those related to the soil/compost environments or to the microbial populations that could be impacted by the presence of nanoparticles.

## 2. Materials and Methods

### 2.1. Materials

Poly(lactic acid) resin (Ingeo^TM^ 2003D) was obtained from NatureWorks LLC. (Minnetonka, MN, USA) with 3.8–4.2% D-LA, number average molecular weight (*Mn*) of 121.1 ± 7.5 kDa, polydispesity index (PDI) of 1.9 ± 0.1, and melt flow index (MFI) of 6 g/10 min (210 °C, 2.16 kg). Cellulose powder (particle size ~20 μm) and Halloysite nanotubes (HNT) were purchased from Sigma-Aldrich (St. Louis, MO, USA). Organo-modified montmorillonite (OMMT) (Cloisite^®^ 30B) and Laponite^®^ RD (LRD) were acquired from BYK Additives Inc. (Gonzales, TX, USA). Additionally, Tomamine^TM^ Q-T-2 (QAC) with 60–70% purity of a methyl, tallow, bis-2-hydroxyethyl, quaternary ammonium, the organo-modifier of Cloisite^®^ 30B, was obtained from Air Products and Chemicals Inc. (Butler, IN, USA). Tetrahydrofuran (THF) was obtained from Pharmco-AAPER (North East, CA, USA). The composition per liter of the R2 broth (R2B) used was 0.5 g yeast extract, 0.5 g proteose peptone #3, 0.5 g casamino acids, 0.5 g dextrose, 0.5 g soluble starch, 0.3 g sodium pyruvate, 0.3 g dipotassium phosphate, and 0.05 g magnesium sulfate. The composition per liter of the M9 minimal medium was 12.8 g Na_2_HPO_4_·7H_2_O, 3 g KH_2_PO_4_, 0.5 g NaCl, 1 g NH_4_Cl, and 1 g of 1 mM MgSO_4_, 1 mM CaCl_2_, 3 × 10^−9^ M (NH_4_)_6_Mo_7_O_24_·4H_2_O, 4 × 10^−7^ M H_3_BO_3_, 3 × 10^−8^ M CoCl_2_·6H_2_O, 1 × 10^−8^ M CuSO_4_·5H_2_O, 8 × 10^−8^ M MnCl_2_·4H_2_O, 1 × 10^−8^ M ZnSO_4_·7H_2_O, 1 × 10^−6^ M FeSO_4_·7H_2_O. All the chemicals and reagents were commercial products of the highest available grade.

### 2.2. Processing of the PLA Bio-Nanocomposites

PLA-BNCs (PLA-OMMT, PLA-LRD, and PLA-HNT) were produced in a two-step process. First, masterbatches were prepared in a ZSK 30 twin-screw extruder (Werner Pfleiderer, NJ, USA) and pelletized. Second, PLA-BNC films (1 and 5 wt % nanoclay) were produced in a cast film microextruder model RCP-0625 (Randcastle Extrusion Systems, Inc., Cedar Grove, NJ, USA). Two PLA-QAC films (0.4 and 1.5 wt % organo-modifier) were produced in a similar fashion. Three PLA films (PLA1, PLA2, and PLA3) with different molecular weight were obtained by varying the processing conditions, and used as control films. In all cases, the materials were dried at 60 °C for 8 h under vacuum (85 kPa) prior to processing. The thickness of the films was measured using a digital micrometer (Testing Machines Inc., New Castle, DE, USA). More details regarding the film processing are provided in Table S1.

### 2.3. Characterization of the PLA Bio-Nanocomposites

To evaluate the presence and dispersion of the nanoclays in the PLA matrix, X-ray diffraction (XRD) and transmission electron microscopy (TEM) were performed. PLA and BNC films were embedded in paraffin blocks and microtomed in 100-nm sections for bright field imaging using an Ultramicrotome MYX (RMC Boeckeler Instruments, Tucson, AZ, USA). TEM micrographs were obtained using a JEOL 2200FS transmission electron microscope (JEOL USA, Inc., Peabody, MA, USA) operating at an acceleration voltage of 200 kV. XRD analysis was conducted in a Rigaku Rotaflex Ru-200BH X-ray diffractometer equipped with a Ni-filtered Cu Kα radiation source setting at 45 kV and 100 mA. The interlayer spacing was calculated according to Bragg’s Law [46]. The carbon, hydrogen and nitrogen content, as well as the amount of nanoclay present in each BNC film was determined by elemental analysis (CHN) and are reported in Table S2. Additional methodologies, such as differential scanning calorimetry (DSC), thermal gravimetric analysis (TGA), moisture isotherm, electrical conductivity, and contact angle, used for characterization of the BNCs are provided in Section S2.

### 2.4. Biodegradation Evaluation

The aerobic biodegradation of PLA and BNCs was evaluated through a series of experiments (Table 1) by analysis of evolved CO_2_ under controlled composting conditions (at 58 °C), using an in-house built direct measurement respirometer (DMR) with a CO_2_ non-dispersive infrared gas analyzer (NDIR). Manure compost from the MSU Composting Facility (East Lansing, MI, USA) was used. The compost was sieved on a 10 mm screen and preconditioned at 58 °C for three days prior to use. Deionized water was incorporated to adjust the moisture content to about 50%. Saturated vermiculite premium grade (Sun Gro Horticulture Distribution Inc., Bellevue, WA, USA) was mixed with the compost (1:4 parts, dry wt. compost) for better aeration. Compost samples were sent to the Soil and Plant Nutrient Laboratory at Michigan State University (East Lansing, MI, USA) for determination of the physicochemical parameters (dry solids, volatile solids, C/N ratio, and pH) and are reported in Table S6. Detailed information about the methods used for compost characterization can be found elsewhere [47].

The bioreactors were loaded with 400 g of compost (or vermiculite) and mixed thoroughly with 8 g of polymer sample (unless otherwise specified). Film samples were cut to 1 cm^2^ pieces and triplicates of each test material were analyzed. Additionally, triplicates of blank bioreactors (with compost or vermiculite only) were evaluated. To simulate composting conditions, the bioreactors were placed in an environmental chamber set at a constant temperature of 58 ± 2 °C. Water-saturated CO_2_-free air was provided to each bioreactor with a flow rate of 40 ± 2 sccm (cm^3^/min at standard temperature and pressure). The bioreactors were incubated in the dark for at least 45 d or until the evolved CO_2_ reached a plateau. For all the biodegradation studies, the results are presented as average (*n* = 3) and standard deviation.

### 2.5. Size Exclusion Chromatography (SEC)

The number average molecular weight (*Mn*), weight average molecular weight (*Mw*), and polydispersity index (*PDI*) of PLA and BNCs before and during composting were determined by SEC with a system from Waters Inc. (Milford, MA, USA) as previously described [47]. Shortly, 20 mg of films were dissolved in 10 cm^3^ of THF and filtered with a hydrophobic polytetrafluoroethylene (0.45 μm pore size) filter. Then, 100 μL of each sample solution were injected. A third-order polynomial calibration curve was obtained from polystyrene (PS) standards ranging 0.5–2,480 kDa, and the Mark–Houwink constants, *K* = 0.000164 dL/g and *α* = 0.704, for PS were used.

### 2.6. Microbial Attachment

*Biofilm Assay*: The biofilm forming ability of microorganisms on the surface of PLA and BNCs was assessed with a biofilm assay in 24-well polystyrene plates as described elsewhere [48,49]. For this test, sterilized PLA films and BNC films were added to the wells of a microtiter plate (24 wells). The films were sterilized by rinsing with 70% ethanol, followed by irradiation with ultraviolet light for 5 min prior to testing. Four replicates of each sample were tested. Each well contained 600 μL of R2B and 200 μL of compost extract (CE), which was prepared by vigorously mixing dry compost with deionized water (1:2 wt./vol.) on vortex for 2 min. The mix was allowed to settle for 20 min and then the supernatant was passed through a sieve with 1 mm mesh. A sterile compost extract (SCE) was prepared for a control by passing the CE twice through a 0.22 μm filter. The inoculated plates were incubated for 48 h at 58 °C gently shaking at 100 rpm. *Pseudomonas aeruginosa* (PA) strain PAO1, a biofilm producing bacterium, was used as a positive control at 23 °C, and uninoculated wells were considered as a negative control. To determine the level of biofilm formed on the surface of PLA and BNCs after incubation, the films were transferred to clean Eppendorf tubes and treated in parallel with the microtiter plates. The broth was removed from the plates and the wells and films were gently washed with water three times. The biofilm was stained with 800 μL of 0.5% crystal violet for 15 min followed by washing three times with water. After the plates and films had air-dried, 800 μL of 30% acetic acid were added, followed by incubation for 15 min. Measurements were done using an Epoch™ Microplate Spectrophotometer (BioTek Instruments, Inc., Winooski, VT, USA) at 600 nm directly on the wells and following decantation of the films. Decanted acetic acid from films was transferred into clean microtiter plates for absorbance measurement at 600 nm. The biofilm formation was quantified by subtracting the average absorbance of the cognate controls from the average absorbance of the inoculated samples.

*Scanning Electron Microscopy (SEM)*: Similar to the biofilm test, sterilized PLA films and PLA-LRD5 films were added to an Erlenmeyer flask containing R2B (2×) and an overnight culture of the compost extract (CE) on R2B at 58 °C (3:1 vol.). The samples were incubated for 48 h at 58 °C. The films were removed from the flasks, gently washed with water three times, and air-dried. The samples were mounted on aluminum stubs using high vacuum carbon tabs (SPI Supplies, West Chester, PA, USA), and coated with osmium. SEM micrographs were obtained at various magnifications using a JEOL 6610LV (tungsten hairpin emitter) scanning electron microscope (JEOL Ltd., Tokyo, Japan) operating at a voltage of 10 kV to observe the biofilm formation.

### 2.7. Statistical Analysis

All statistical analyses were performed using Minitab18 software (Minitab Inc., State College, PA, USA) by analysis of variance (one way ANOVA), and Tukey test with a *p*-value threshold of 0.05 as for level of significance. Data are reported as mean and standard deviations.

## 3. Results and Discussion

### 3.1. Characterization of the PLA Bio-Nanocomposites

**Figure 1:**
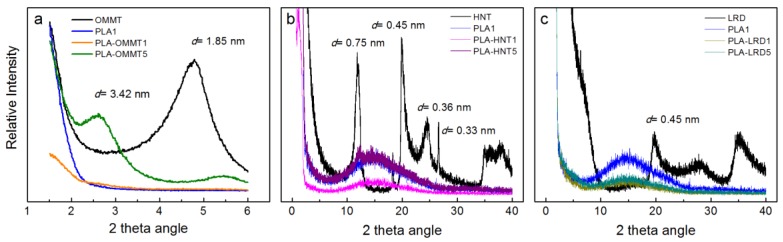
XRD spectra of the different nanoclays, PLA1, and (**a**) OMMT, (**b**) HNT, and (**c**) LRD bio-nanocomposite films.

**Figure 2:**
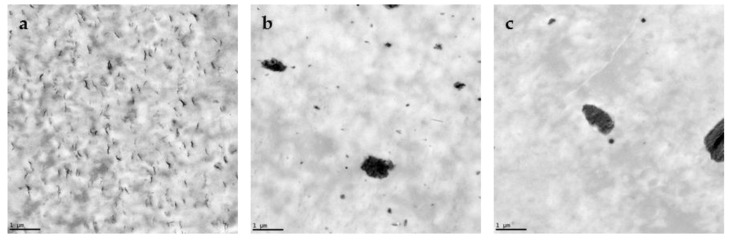
TEM micrographs of (**a**) PLA-OMMT5, (**b**) PLA-HNT5, and (**c**) PLA-LRD5 bio-nanocomposites at 10k×. The bar in the left bottom represents 1 μm.

Figure 1 and Figure 2 show the XRD spectra and TEM micrographs of the BNCs, respectively. These methods were used to evaluate the presence and dispersion of the nanoclays in the PLA matrix. Depending on the degree of dispersion, a layered silicate nanocomposite can be either intercalated or exfoliated. Intercalation occurs when the polymer chains penetrate into the interlayer regions of the clay, while exfoliation is observed when the clay layers are delaminated and randomly dispersed in the polymer matrix [3]. As observed in Figure 1a, in the case of PLA-OMMT5 film, OMMT is not fully exfoliated but intercalated in the PLA matrix, which is represented by the shift of the peak to the left, i.e., the increase in the interlayer distance from 1.85 nm, for the pristine OMMT, to 3.42 nm, for the OMMT present in the film. The organic modification of the MMT through exchange of cationic ions allows for better dispersion and exfoliation of the silicate layers into the PLA matrix [1,3,7]. However, in the case of PLA-OMMT5 it was not enough to obtain a fully exfoliated BNC. This was confirmed by the TEM micrograph (Figure 2a), which shows some small agglomerations. However, it seems that the OMMT is evenly distributed in the PLA matrix. PLA-OMMT1 showed a better dispersion of the OMMT in the polymer matrix, but in general, full exfoliation is difficult to achieve, and most nanocomposites are a mixture of both structures, which is usually referred to as disordered morphology or orderly exfoliated morphology [4].

Similarly, Figure 1b,c show the XRD spectra of HNT and LRD nanocomposites, respectively. In both cases, the profiles showed broad peaks around a 2θ angle of 16, which are representative of amorphous PLA samples [50,51]. HNT is an alumina-silicate clay with an elongated hollow tubular structure consisting of an external surface composed of siloxane (Si-O-Si) groups and an inner side and edges consisting of (Al-OH) groups [16,24,52]. In the XRD spectrum of the HNT nanoclay (Figure 1b), the presence of three main peaks at 2θ angles of 12.02, 19.99, and 24.54 can be observed, corresponding to the basal *d*-spacing of 0.75, 0.45, and 0.36 nm, respectively. Similar diffraction patterns are reported elsewhere [24,53,54,55,56,57]. In the case of PLA-HNT5, the presence of a peak at 2θ angle of 12.25 was observed. The small shift to the right, from the 12.02 of the pristine HNT, indicates a reduction in the *d*-spacing. This behavior has been observed by other researchers, and was attributed to the formation of a micro-filled composite [24,54]. The disappearance of the other peaks, such in the case of PLA-HNT5 and PLA-HNT1, has been explained as due to the interaction of the polymer chains with the nanotubes, and also due to the preferential orientation of nanotubes during processing of the film [19,24]. It was also observed that the intensity of the characteristic peaks depends on the level of loading of nanoclay [53,54].

LRD particles have a disk-like shape with two external tetrahedral silica sheets that present continuous corner-shared tetrahedral SiO_4_ units arranged in hexagonal rings, and a central octahedral magnesia sheet that is composed of bivalent or trivalent cations sharing the edges coordinated to hydroxyl groups. The excess negative charge is compensated by the presence of Na ions between the silicate layers [25,27,28,29]. In the XRD spectrum of the LRD nanoclay (Figure 1c), the presence of the characteristic LRD peak at 2θ angle of 19.8 can be observed, corresponding to the basal *d*-spacing of 0.45 nm. Similar diffraction patterns are reported for LRD elsewhere [25,26]. In the XRD spectra of the PLA-LRD, no diffraction peaks were observed. This behavior has been attributed, in the literature, to separated LRD platelets dispersed individually in the polymer matrix [25]. The nanoclay dispersion was also confirmed by TEM.

Figure 2b,c show the TEM micrographs of HNT and LRD nanocomposites, respectively. In the case of PLA-HNT5, Figure 2b shows the presence of big agglomerations indicating that HNT was not evenly distributed in the PLA matrix. Similar observations have been reported in the literature for PLA-HNT nanocomposites [20,53]. A similar distribution was also found for the PLA-LRD5 film (Figure 2c).

Other factors influencing the nanoclay dispersion in the PLA matrix are the level of loading and the size of the nanoparticles [26]. For example, HNT and LRD are bigger particles than MMT. While MMT has layers with 1 nm thickness and tangential dimensions from 300 Å to a few microns [1,3,7], HNT has inner and outer diameters of the tube ranging from 10 nm to 40 nm and 40 nm to 70 nm, respectively, while the length ranges from 0.2 μm to 3 μm [16,24,52]. LRD usually has dimensions around 25–30 nm in diameter and 1 nm in thickness [26,27,29].

### 3.2. Biodegradation Evaluation

**Figure 3:**
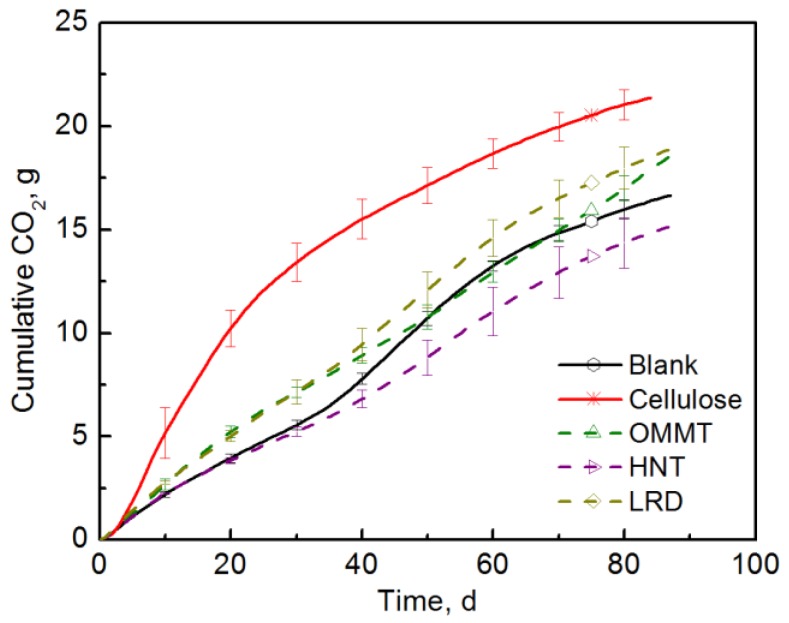
CO_2_ evolution of the three different nanoclays (Test I in compost).

**Figure 4:**
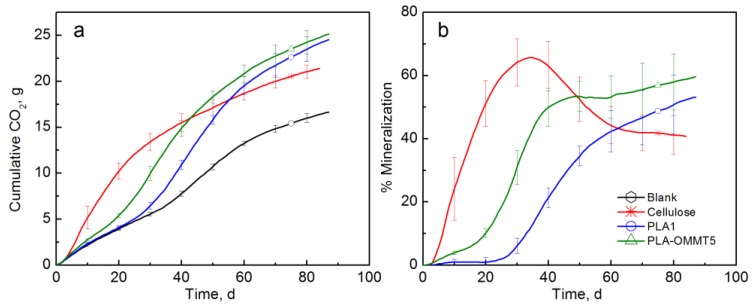
(**a**) CO_2_ evolution and (**b**) % Mineralization of PLA and PLA-OMMT5 films (Test I in compost).

**Figure 5:**
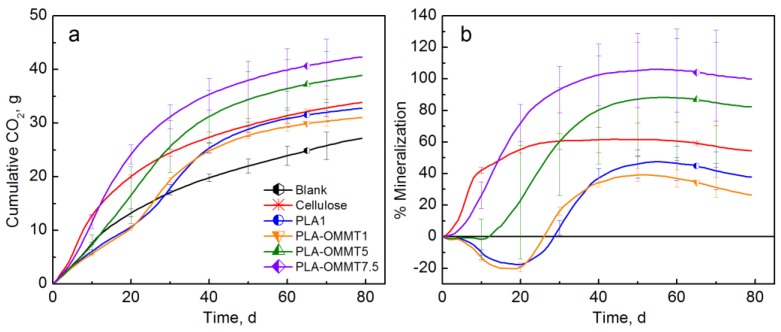
(**a**) CO_2_ evolution and (**b**) % Mineralization of PLA and PLA-OMMT films with three different levels of loading (1, 5, and 7.5%) (Test II in compost).

**Figure 6:**
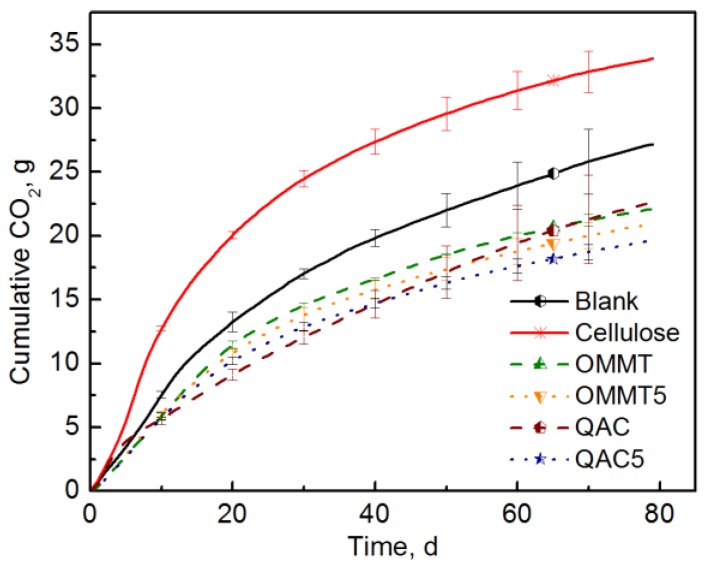
CO_2_ evolution of OMMT nanoclay and QAC surfactant (Test II in compost).

**Figure 7:**
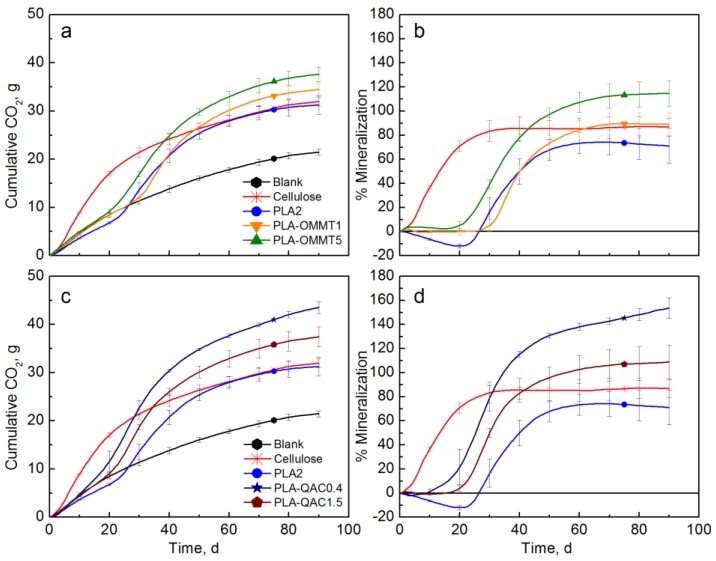
CO_2_ evolution and % Mineralization of PLA-OMMT films (**a**,**b**) and PLA-QAC films (**c**,**d**) (Test III in compost).

**Figure 8:**
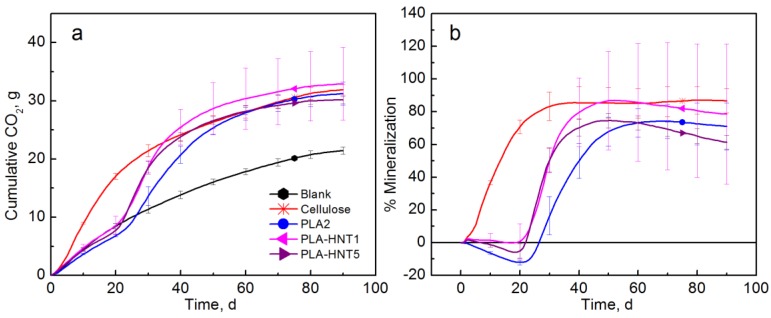
(**a**) CO_2_ evolution and (**b**) % Mineralization of PLA-HNT films (Test III in compost).

**Figure 9:**
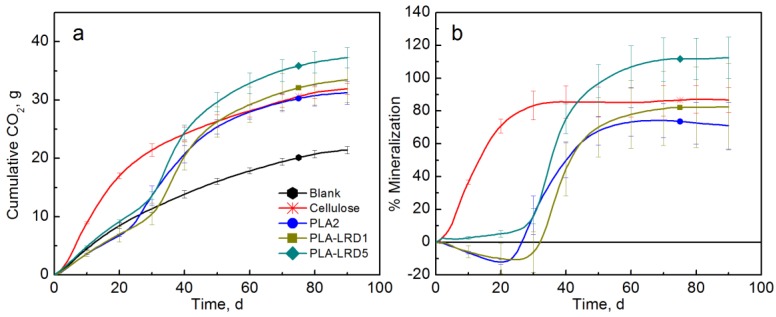
(**a**) CO_2_ evolution and (**b**) % Mineralization of PLA-LRD films (Test III in compost).

**Figure 10:**
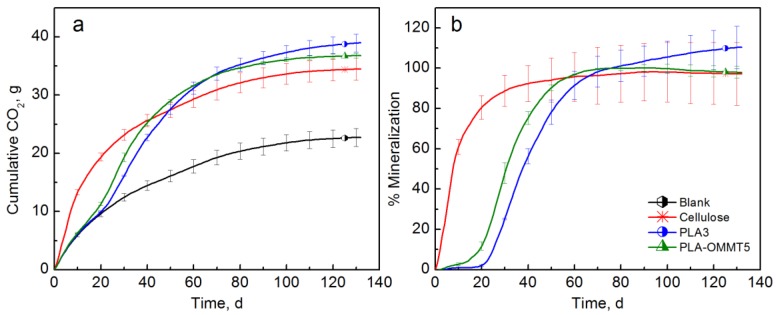
(**a**) CO_2_ evolution and (**b**) % Mineralization of PLA and PLA-OMMT5 films (Test IV in compost).

**Figure 11:**
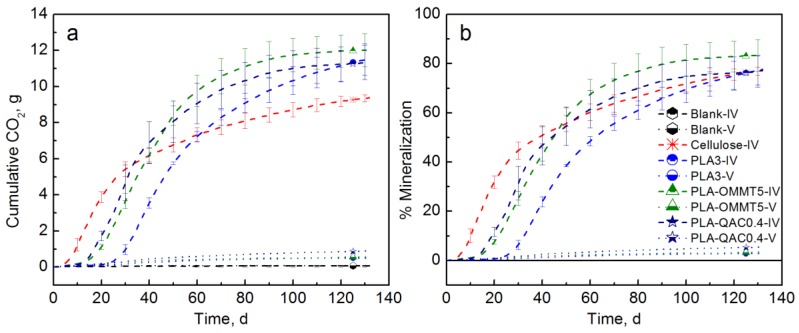
(**a**) CO_2_ evolution and (**b**) % Mineralization of PLA, PLA-OMMT5, and PLA-QAC0.4 (Test IV in inoculated vermiculite (dashed lines) and uninoculated vermiculite (dotted lines)).

The DMR system was used to perform four different biodegradation tests in which the CO_2_ evolved from each bioreactor was measured with controlled temperature, RH, and air flow rate. For the data analysis, the average cumulative CO_2_ and % mineralization of each test material was calculated and plotted as a function of time. Detailed information about the concepts and calculations is provided elsewhere [47,58,59,60]. The blank bioreactors contain the solid media only (i.e., compost or vermiculite). In all cases, cellulose powder was used as a positive reference material since it is a well-known easily biodegradable material. The cumulative CO_2_ and % mineralization curves obtained from the different biodegradation tests for the evaluation of PLA and PLA-BNCs, as well as the different nanoclays and surfactant, are presented in Figure 3, Figure 4, Figure 5, Figure 6, Figure 7, Figure 8, Figure 9, Figure 10 and Figure 11.

To evaluate the effect of the nanoclays on the compost microbial population, the three different nanoclays were tested in the powder form as received. Figure 3 shows the CO_2_ evolved from the bioreactors containing the three different nanoclays. A significant difference between the CO_2_ evolved from cellulose and the one from the nanoclays was observed. During the first 40 days of the test, OMMT and LRD bioreactors produced a significantly higher amount of CO_2_ than the blank indicating that there was no inhibition. On the contrary, the HNT bioreactors produced equal or less CO_2_ than the blank, especially after 35 days, indicating some kind of inhibition in which HNT may limit the availability and/or the distribution of carbon and other nutrients for basic microorganism functions.

Figure 4 shows the CO_2_ and % mineralization of the pristine PLA film and PLA-OMMT5. The typical PLA biodegradation behavior with the presence of a lag time of around 25 days was observed [47,61]. The lag time observed in the biodegradation of PLA has been explained by the low diffusion rate of the byproducts formed during the hydrolytic degradation and present inside the sample [62]. Cellulose reached a maximum mineralization of 65.7% after 34 days while PLA and PLA-OMMT5 reached 53.2 and 59.6% after 87 days, respectively. The decrease in the mineralization curve of cellulose indicates that these bioreactors were no longer producing more CO_2_ than the blank bioreactors. This behavior may be explained by a rapid and large increase of the microbial population at the beginning of the test when there are plenty of resources easily available for microbial assimilation. Then, a decrease in the mineralization curve is observed when these resources are depleted and/or limited [47]. Even though by the end of the test, the mineralization of PLA and PLA-OMMT5 was not significantly different, it was clearly observed that the lag phase of the pristine PLA was longer than the PLA-OMMT5. The mineralization of PLA-OMMT5 was significantly higher before day 60. Among the different explanations for this accelerated biodegradation due to OMMT found in the literature is the relatively high hydrophilicity of the nanoclay, which improves the diffusion of water into the PLA polymeric matrix and in turn promotes hydrolytic degradation [33,37,38,44,62]. Another reason is that the presence of terminal hydroxyl groups in the silicate layers and in some organo-modifiers promotes the hydrolytic degradation of PLA [10,44,63]. However, the molecular weight of the PLA-OMMT5 films and the thickness can also play a crucial role and influence the observed results [47].

To evaluate the effect of clay loading on the biodegradation of PLA, three films with different loadings of OMMT (1, 5, and 7.5 wt %) were tested. Figure 5 shows the CO_2_ evolution and % mineralization of PLA and PLA-OMMT films. Cellulose reached a maximum mineralization of 61.7% after 45 days of testing. The biodegradation behavior of the pristine PLA and PLA-OMMT1 was similar, again with a typical lag time at the beginning of the biodegradation test. The negative mineralization values observed in Figure 5b are generated as an artifact when the blank bioreactors produce more CO_2_ than the sample material bioreactors. This effect might be caused because of the physical barrier offered by the polymer film at this early stage of the test, contrary to the PLA-OMMT5 and PLA-OMMT7.5 in which their biodegradation phase started much earlier. The observed shorter lag time of PLA-OMMT5 is in agreement with the previous test results, but in this case the average mineralization was significantly higher than the PLA control. It seems that PLA-OMMT7.5 has the highest average mineralization and the fastest biodegradation rate in which the lag time was only around five days. However, mineralization values above 100% indicate the presence of a priming effect, in which the additional carbon converted to CO_2_, is not coming from the sample material but from the over-degradation of the indigenous organic carbon present in the compost [47,64]. Again, the initial molecular weight of the films should influence the observed results. It is important to mention that during the processing of the films, with different nanoclay loading, the resulting molecular weight was affected even though, in this case, the same processing conditions were maintained, with the higher clay loading corresponding to the lower molecular weight. Furthermore, Roy et al. analyzed the water-soluble degradation products by electrospray ionization-mass spectrometry (ESIMS), and their results indicated a catalytic effect of MMT in hydrolysis of PLA since shorter lactic acid oligomers were formed in the case of PLA/MMT composites [41]. Some researchers have attributed a plasticizing effect to the degradation byproducts (i.e., lactic acid oligomers and monomers), represented by a decrease in the *T_g_* of PLA and BNCs. In this context, faster biodegradation of the PLA and BNC could also be induced by the increased segmental mobility of backbone chains and the expanded amorphous regions of the polymeric matrix [44,62,65]. Another factor influencing the biodegradation rate of the BNCs is the crystallinity of the material. The presence of nanoclays could affect the degree of crystallization of PLA (Table S3), with the amorphous parts preferentially biodegrading [47].

The effect of the amount/concentration of clay and surfactant on the compost microbial populations was evaluated and the results are shown in Figure 6. In this case, OMMT refers to 8 g of the tested sample material, while OMMT5 refers to the theoretical amount of nanoclay contained in 8 g of PLA-OMMT5 film. Similarly, QAC refers to 8 g of the tested sample material and QAC5 to the theoretical amount of surfactant contained in 8 g of PLA-OMMT5 film. Regardless of the concentration of either OMMT or QAC, the CO_2_ evolution was always significantly lower than the blank, indicating that there was clear inhibition of the microbial activity when these materials were present by themselves.

Figure 7 shows the results of a different biodegradation test in which the PLA-OMMT and the PLA-QAC films were evaluated. Cellulose reached a mineralization of 85.5% after 38 days of testing, while the PLA control reached 74.2% after 69 days. As in the previous test, there was no significant difference between the pristine PLA and the PLA-OMMT1 films (Figure 7b). However, PLA-OMMT5 had significantly higher mineralization and a shorter lag time than the PLA control. A priming effect was observed with mineralization values over 100%. The PLA films containing the surfactant (QAC) also showed reduced lag time and a significantly higher amount of evolved CO_2_ than the PLA control, and in both cases a priming effect was observed (Figure 7d). This may be due to the lower initial molecular weight of these films. In our previous work [47], it was demonstrated that the PLA film with the lowest *M_n_* presented a priming effect when tested in compost, but it was not observed in inoculated vermiculite, having mineralization values closer to the other two tested PLA films with higher *M_n_*. PLA-OMMT5 and PLA-QAC0.4 were also tested in inoculated and uninoculated vermiculite, and the results are later shown in Figure 11. Similarly, the priming effect was not observed in this case.

Figure 8 shows that the mineralization of PLA-HNT films was not significantly different from the PLA control by the end of the test (90 days). However, it can be clearly observed that with both levels of loading the lag time was reduced and the mineralization was significantly different before day 45. A higher variability and also a priming effect were observed in the biodegradation of PLA-HNT1 film. PLA-HNT films reached their maximum mineralization after 50 days of testing with an average of 86.9 and 74.6% for PLA-HNT1 and PLA-HNT5, respectively.

As observed in Figure 9, PLA-LRD5 showed significantly higher mineralization than the pristine PLA and the PLA-LRD1 films. In this case, the lag time was not reduced but the PLA-LRD5 showed a priming effect. PLA-LRD films reached their maximum mineralization by the end of the test with an average of 82.5 and 112.5% for PLA-LRD1 and PLA-LRD5, respectively.

To avoid the priming effect observed in the previous tests, a specific new biodegradation test was performed in three different solid environments (compost, inoculated vermiculite, vermiculite) as described elsewhere [47]. When tested in compost (Figure 10), there was no significant difference in the mineralization of these materials by the end of the test (132 days). However, it seems that the mineralization of PLA-OMMT5 was significantly higher than the PLA during the first 45 days of testing. Similarly to the previous tests, PLA-OMMT5 showed a reduced lag time and a priming effect could be occurring due to the low molecular weight of both films. The maximum average mineralization for PLA and PLA-OMMT5 was 110.4 and 100.2%, respectively.

The biodegradation test with inoculated vermiculite should avoid the priming effect as previously demonstrated [47,64,66]. Figure 11 shows that there was no significant difference in the mineralization of the tested materials at the end of the test (132 days). However, both PLA-OMMT5 and PLA-QAC0.4 showed significantly higher mineralization than the PLA control before 70 days of testing, and a much shorter lag time. The PLA control reached 77.7% mineralization after 132 days while PLA-OMMT5 reached the same mineralization after 83 days of testing and a maximum average mineralization of 83.3%. PLA-QAC reached a mineralization of 77.3%. It is important to mention that longer testing times were expected in this case since the biodegradation in inoculated vermiculite occurs at a slower rate than in compost. Even though the initial molecular weight of the films has a strong effect on their mineralization and priming effect, it seems that the addition of OMMT also accelerated the initial degradation of the samples. As previously mentioned, this behavior may be explained by the improved diffusion of water into PLA due to the high hydrophilicity of the nanoclay, which in turn promotes hydrolytic degradation [33,37,38,44,62].

Figure 11 also shows the results when testing with uninoculated vermiculate. As expected, there was no significant evolution of CO_2_ in the abiotic degradation test, and there was no significant difference in the mineralization values. For the biodegradation test III, film samples were taken at different periods of time in order to track the changes in the molecular weight and the results are explained in Section 3.3.

### 3.3. Molecular Weight

**Figure 12:**
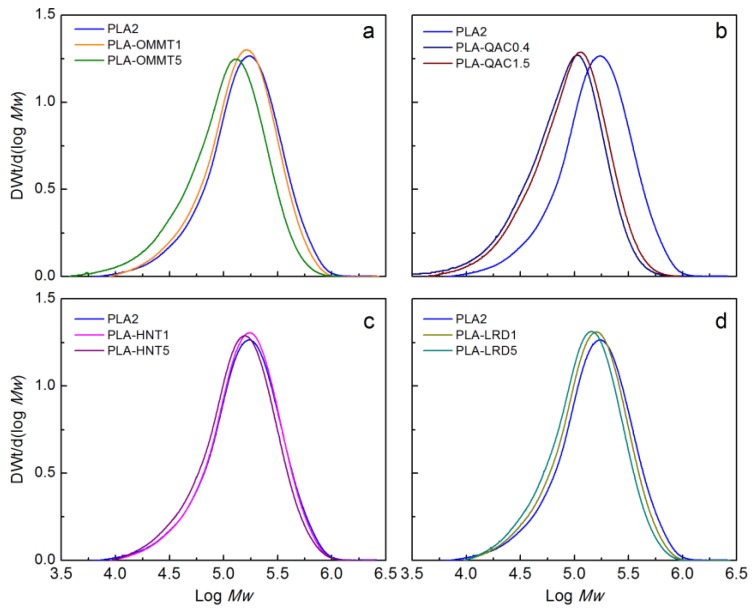
Initial molecular weight of PLA and BNCs.

Figure 12 shows the initial molecular weight distribution (MWD) of the PLA film and BNCs. As previously mentioned, the addition of nanoclay resulted on a reduction of the *M_n_* during processing. This reduction in *M_n_* was more pronounced in the case of PLA-OMMT5, PLA-QAC1.5, and PLA-QAC0.4. More detailed information about the initial *M_n_, M_w_*, and *PDI*, of PLA and BNCs films is provided in Table S7.

**Figure 13:**
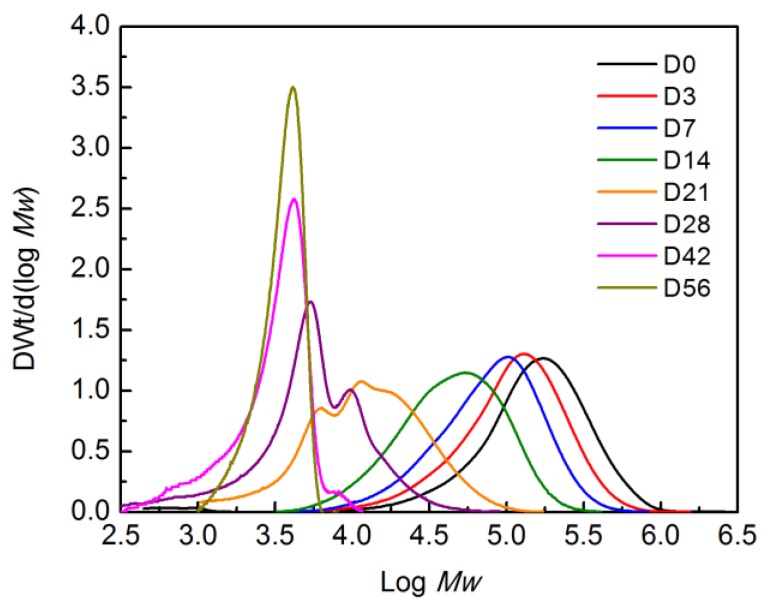
Change in molecular weight of PLA2 film (Test III in compost).

Figure 13 shows the decrease of molecular weight of the PLA control film as a function of time during the biodegradation test III, represented by the shift of the peak to the left. This behavior was previously reported in the literature during the hydrolytic degradation of PLA, and was attributed to the chain scission preferentially occurring in the bulk of the polymer matrix rather than the surface [67]. The broadening of the peaks over time indicates an increase in the *PDI* due to the fragmentation of the PLA chains. The change in the MWD from monomodal to multimodal after day 14 has also been previously observed during hydrolytic degradation of PLA and was attributed to the formation of crystalline residues due to the rearrangement of the new shorter polymer chains into a more stable configuration (i.e., crystalline structures) [51,67]. The formation of more defined and higher peaks, as observed at days 42 and 56, has been attributed to the predominant degradation of the amorphous regions [68]. During the biodegradation tests a whitening effect in PLA and BNC was observed. It has been reported that this effect indicates increased crystallinity and opacity due to the beginning of the hydrolytic degradation phase of the biodegradation process [44,45,62]. The whitening effect occurs because a change in the refraction index of the polymer is induced by the absorbed water and/or the byproducts, e.g., carboxylic end-groups that are able to catalyze ester hydrolysis [45,62].

**Figure 14:**
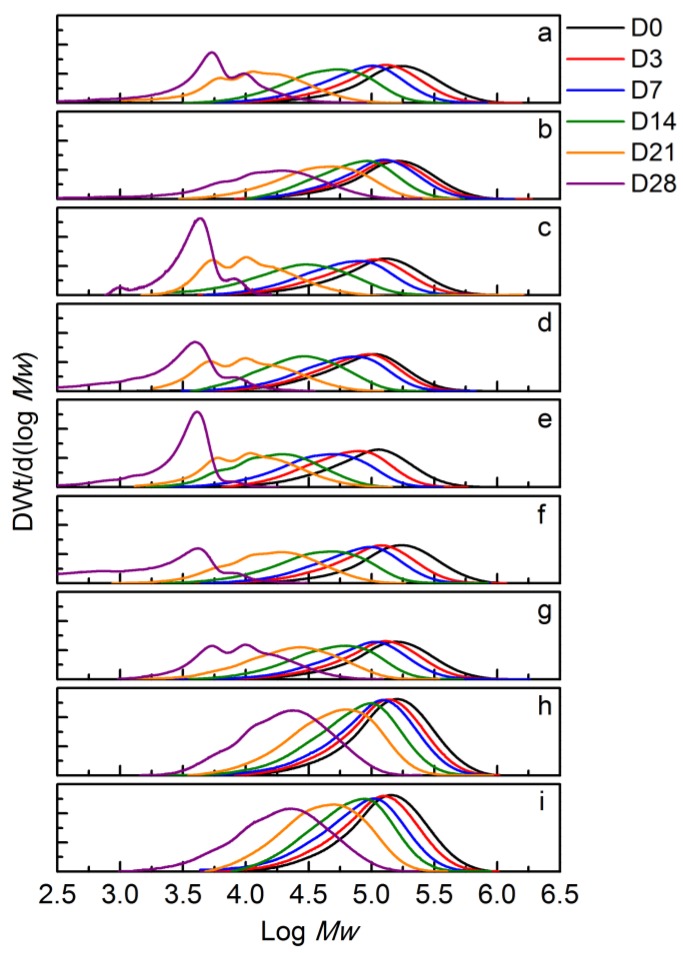
Change in molecular weight of (**a**) PLA2, (**b**) PLA-OMMT1, (**c**) PLA-OMMT5, (**d**) PLA-QAC0.4, (**e**) PLA-QAC1.5, (**f**) PLA-HNT1, (**g**) PLA-HNT5, (**h**) PLA-LRD1, and (**i**) PLA-LRD5 films (Test III in compost).

Figure 14 shows the changes in the MWD of the BNCs as function of time until day 28 since it was not possible to collect samples for SEC analysis after that period of time (except for PLA control as shown in Figure 13). Similarly to the PLA control, the BNCs showed multimodal peaks after day 14, although more evidently after day 21. In general, this behavior was less pronounced for PLA-OMMT1, PLA-LRD1, and PLA-LRD5, and it may be attributed to a slower formation of crystalline residuals. From Figure 14, it can be observed that the reduction of molecular weight was slower for PLA-OMMT1 and PLA-LRD1, in comparison with the pristine PLA. Similarly, the MWD of PLA-OMMT5 and PLA-QAC15 have a similar trend with an evident multimodal peak at day 21, while the reduction of molecular weight of PLA-HNT5 and PLA-LRD5 films seems to be slower than PLA control.

Deconvolution of the peaks was performed due to the multimodal MWD observed in the previous results, followed by kinetics analysis (Section S4). The *M_n_* reduction rate (*k*) constant was calculated for PLA and the BNCs, fitting of a first order reaction of the form *M_n_*/*M_n_*_0_ = exp(−*kt*), where *M_n0_* is the initial *M_n_*, *k* is the rate constant, and *t* is the time. The results (Figure S6 and Table S8) show that the BNCs, especially PLA-LRD films, have a lower *M_n_* reduction rate than the PLA control (*k* = 0.1008 ± 0.0037) until day 28. Ray and Okamoto analyzed the molecular weight of PLA and PLA nanocomposites and found that the reduction was almost the same for all the samples [10]. In contrast, Paul et al. found that the *M_n_* of PLA decreased ~40% with respect to its initial value while for the PLA nanocomposites *M_n_* decreased 70–80% [38]. In this case, even though the *M_n_* reduction rate of the BNC was the same or lower than the PLA control, a higher evolution of CO_2_ from the bioreactors supplemented with the BNC was generally observed during the biodegradation tests. Therefore, it is also relevant to understand the role of the microorganisms and how they are affected by the presence of these nanoclays. For example, Annamalai et al. suggested that the clay nanoparticles improve the absorption of UV light and promote polymer photo-oxidation due to the catalytic effect of metal ion impurities. That increased oxidation at the surface of the nanocomposites could favor the adhesion, accumulation and growth of the microorganisms [69].

### 3.4. Microbial Attachment

Biofilm assays were performed to evaluate the ability of the microorganisms present in the compost to attach to the surface of PLA film and BNCs (i.e., PLA-OMMT5, PLA-QAC0.4, PLA-HNT5, and PLA-LRD5). Even though biofilm formation does not necessarily mean that the material is biodegraded by the attached populations [70], it is an important aspect of microbial performance and survival [71]. When biofilm-forming microorganisms release exopolymeric substances (EPS) (e.g., carbohydrates, nucleic acids, and proteins) such resources become available for other microorganisms, including secreted enzymes that degrade PLA and derivatives. Secreting extracellular digestive enzymes after forming a biofilm would localize the effect of extracellular digestion and increase the benefit to biofilm-forming strains. Biofilm production is a common trait among microorganisms living in soil, which are usually exposed to low moisture conditions. Biofilms can contribute to water retention in the soil matrix, prevent microorganisms from being washed out, and confer tolerance to other environmental stressors [71].

**Figure 15:**
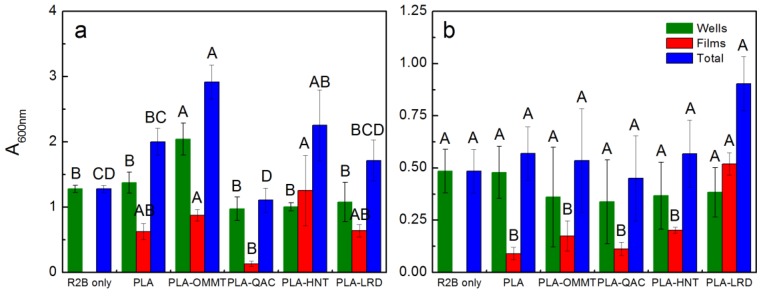
Absorbance (600 nm) of (**a**) PA at 23 °C, and (**b**) CE at 58 °C for second biofilm test. Columns with the same letter within a group (i.e., wells, films, or total) are not significantly different at *p* ≤ 0.05 (Tukey test).

An initial test of the biofilm assay is shown in the Supplementary Materials (Section S5). Figure 15 and Tables S11 and S12 show the results of the biofilm test. A positive control was performed using *Pseudomonas aeruginosa* (PA) strain PAO1, a high biofilm forming strain, at 23 °C [72,73]. Looking at the control with PA at 23 °C (Figure 15a), it was observed that the positive control wells (PA + R2B) had an absorbance (600 nm) of 1.226–1.332, with uninoculated control wells ranging from 0.060 to 0.065, which is in agreement with the values reported by Satti et al. [49]. The wells containing PLA, PLA-QAC0.4, PLA-HNT5, and PLA-LRD5 were approximately the same as the control lacking any film (R2B only). However, the wells containing PLA-OMMT5 showed significantly more biofilm formation (average 2.042), suggesting that the OMMT had an indirect stimulation on biofilm formation by PA. For the biofilm formed on the surface of the films by PA at 23 °C, PLA ranged from 0.501 to 0.752, which is also in agreement with the values previously observed [49]. In this case, the values of PLA-OMMT5 and PLA-HNT5 were significantly different from PLA-QAC0.4. PLA-HNT5 had one of the highest average values with 1.254. Looking at the total biofilm formation, PLA-OMMT5 and PLA-QAC0.4 were significantly different from pristine PLA and the rest of the BNCs with the highest (2.917) and lowest (1.107) values, respectively. The total average biofilm values (wells + film) for PA at 23 °C in descending order are as follows PLA-OMMT5 > PLA-HNT5 > PLA > PLA-LRD5 > PLA-QAC0.4.

Regarding the biofilm estimates with CE at 58 °C (Figure 15b), the sterile controls (SCE) have values that are between 0.101 and 0.124, which are slightly greater than what was seen with low nutrient media at 23 °C. This is probably due to significant amounts of humic material in the CE. The control wells (CE only) have values of 0.381–0.588. These values are less than the ones for PA at 23 °C, which is expected since PA is a well-known biofilm former and because microbial growth and survival is generally more challenging at 58 °C and CE contains a diverse collection of microbial populations, many of which do not form biofilm under these conditions. The wells supplemented with PLA and BNCs ranged from 0.122–0.603 with no statistically significant difference among them. Biofilm formation was observed on the surface of PLA and BNCs with CE at 58 °C. PLA-LRD5 has significantly higher value (0.519) than the rest of the BNCs. The lowest average values were observed for PLA-QAC0.4 and PLA with 0.113 and 0.090, respectively. In this case, the total biofilm was also not significantly different among the sample materials.

In general, the PLA-LRD5 biofilm was the largest among the different samples, indicating that population in CE have a preference for PLA-LRD5 at 58 °C. In contrast, a pure culture, *Pseudomonas aeruginosa*, clearly preferred PLA-OMMT5 at 23 °C. Overall the biofilms at 58 °C were smaller than the biofilm at 23 °C. At both temperatures, PLA-QAC0.4 was the film producing the lowest average amount of biofilm, which may be attributed to inhibition due to the surfactant. This is supported by the biodegradation test where the surfactant was tested alone. Further investigation is recommended to understand which specific microbial strains present in the compost bind to and preferentially degrade PLA and the BNCs.

**Figure 16:**
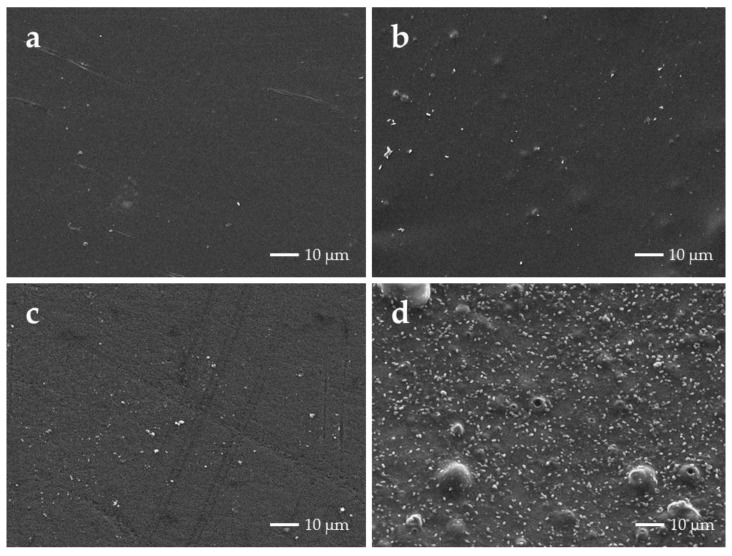
SEM micrographs of (**a**) PLA and (**b**) PLA-LRD at 1000× before incubation, (**c**) PLA and (**d**) PLA-LRD5 after incubation for 48 h at 58 °C with compost extract in R2B.

Due to the significant differences between pristine PLA and PLA-LRD5 found in the biofilm formed on the surface of the films during the test at 58 °C with CE, several SEM micrographs were taken from samples coated with osmium. Figure 16 shows the difference in microbial attachment between pristine PLA and PLA-LRD5 at a magnification of 1000×. It can be clearly observed that the surface of PLA-LRD5 is much more heavily populated by microorganisms, in agreement with the biofilm test results (Figure 15b).

## 4. Conclusions

The effect of three different nanoclays, OMMT, HNT, and LRD, as well as the OMMT organo-modifier (QAC) on the biodegradation of PLA was evaluated with an in-house built DMR system following the analysis of evolved CO_2_ approach. The results obtained from four different biodegradation tests along with the study of microbial attachment on the surface of PLA and its BNCs show that the biodegradation phase of the films containing nanoclay started earlier than that for pristine PLA. This behavior was confirmed by the results obtained from different tests for PLA-OMMT5, even when tested in inoculated vermiculite. The tests performed in vermiculite allowed untangling the observed priming effect even though longer testing times were required. The effect of the nanoclays on the initial molecular weight during processing played a crucial role in the biodegradation studies, also since a lower *M_n_*_0_ (≤60 kDa) seems to be correlated to the priming effect in compost. Further investigation is recommended using PLA and BNCs with the same initial molecular weight and thickness, a task not easy to achieve in lab settings. When the different nanoclays and surfactant were tested alone, it was observed that HNT, OMMT, and QAC showed some inhibition regardless of the amount introduced in the bioreactors. PLA-LRD5 showed a priming effect with mineralization values exceeding 100%. This behavior may be explained by the lower initial molecular weight and by the results observed during the microbial attachment tests, in which PLA-LRD5 showed the greatest biofilm formation on the surface as confirmed by the SEM micrographs. PLA-QAC0.4 had the lowest biofilm formation, which may be attributed to the inhibitory effect also found during the CO_2_ evolution test when QAC was tested alone. Under the experimental conditions used to investigate biofilm formation, it was noted that significant biofilm was established in only 48 h; however, the timing may be different in composting conditions. Further investigation is required on the specific microbial strains that are capable of biodegrading PLA and its BNCs and how they can affect the biodegradation rate. Disposable products like packaging would greatly benefit from the biodegradable features of PLA since it would allow its disposal along with other organic wastes in composting facilities.

## Figures and Tables

**Table 1:** Key for biodegradation test and labels of the samples.

Test ID	Samples Tested
I	Blank, Cellulose, OMMT, HNT, LRD, PLA1, PLA-OMMT5
II	Blank, Cellulose, OMMT, OMMT5, QAC, QAC5, PLA1, PLA-OMMT1, PLA-OMMT5, PLA-OMMT7.5
III	Blank, Cellulose, PLA2, PLA-OMMT1, PLA-OMMT5, PLA-HNT1, PLA-HNT5, PLA-LRD1, PLA-LRD5, PLA-QAC1.5, PLA-QAC0.4
IV	Blank, Cellulose, PLA1, PLA2, PLA3, PLA-OMMT5, PLA-QAC0.4

## Supplementary Materials

Information about material processing and characterization, physicochemical characteristics of the compost, molecular weight determination, and biofilm test formation are available online at www.mdpi.com/link.
