# Supplementary material for: Impact of Nanoclays on the Biodegradation of Poly(Lactic Acid) Nanocomposites
Source: Polymers (Basel). 2018 Feb 17;10(2):202. doi: 10.3390/polym10020202 (PMC6415156; doi:10.3390/polym10020202)
Supplement: Supplementary file 1 [file polymers-10-00202-s001.pdf]

polymers

MDPI

Article

Impact of Nanoclays on the Biodegradation of Poly(Lactic Acid) Nanocomposites

Edgar Castro-Aguirre [1], Rafael Auras [1,*], Susan Selke [1], Maria Rubino [1] and Terence Marsh [2]

[1] School of Packaging, Michigan State University, East Lansing, MI 48824, USA; castroag@msu.edu (E.C.A.); sselke@msu.edu (S.S.); mariar@msu.edu (M.R.)
[2] Department of Microbiology and Molecular Genetics, Michigan State University, East Lansing, MI 48824, USA; marsht@msu.edu (T.M.)
* Correspondence: aurasraf@msu.edu (R.A.); Tel.: +1-517-432-3254

Received: 9 January 2018; Accepted: 12 February 2018; Published: date

Supplementary Materials

Table of Contents

S1: Material processing;

S2: Material characterization;

S3: Physicochemical characteristics of the compost;

S4: Molecular weight determination;

S5: Biofilm formation;

S6: References.

S1: Material processing

Masterbatch (MB) production: The PLA-BNCs (PLA-OMMT, PLA-HNT, and PLA-LRD) masterbatches (15 – 20% nanoclay wt.) were prepared in a ZSK 30 twin-screw extruder (Werner Pfleiderer, NJ) and pelletized. A PLA-QAC masterbatch (10% QAC wt.) was prepared in a similar fashion. Pristine PLA was processed in the twin-screw extruder and used for the processing of PLA1 control film. Table S1 shows the general MB processing conditions.

Film production: All films were produced by using a Microextruder model RCP-0625 (Randcastle Extrusion Systems, Inc., Cedar Grove, NJ) with a screw diameter of 15.9 mm, screw L/D of 24, and volume of 34 cm^3. Table S1 shows the processing conditions of the films and their thickness as measured with a digital thickness micrometer. However, it was observed that the measurement of the BNC's thickness with the digital micrometer may not be the best approach due to the presence of the nanoclay. The thickness of PLA-OMMT1 and PLA-OMMT5 was measured from the SEM cross-section of the films and it was found to be 0.020 ± 0.004, and 0.010 ± 0.002 mm, respectively.

Table S1. Processing conditions of the sample materials.

Material	Concentration, wt%	Type	Temperature range, °C	rpm	Thickness, mm
PLA	0%	MB	146-186	130	N/A
PLA-OMMT	20%	MB	146-186	130	N/A
PLA-QAC	10%	MB	148-189	130	N/A
PLA-HNT	15%	MB	159-181	40	N/A
PLA-LRD	15%	MB	159-181	40	N/A
PLA1	0%	Film	194-216	49	0.031 ± 0.006
PLA2	0%	Film	193-249	33	0.022 ± 0.003
PLA3	0%	Film	193-249	28	0.034 ± 0.009
PLA-OMMT	1%	Film	193-243	18	0.044 ± 0.007
PLA-OMMT	5%	Film	193-248	18	0.073 ± 0.014
PLA-OMMT	7.5%	Film	193-243	18	0.089 ± 0.013
PLA-QAC	0.4%	Film	143-173	31	0.039 ± 0.008
PLA-QAC	1.5%	Film	143-173	31	0.036 ± 0.011
PLA-HNT	1%	Film	193-216	23	0.037 ± 0.007
PLA-HNT	5%	Film	193-216	23	0.050 ± 0.006
PLA-LRD	1%	Film	193-216	23	0.064 ± 0.013
PLA-LRD	5%	Film	193-216	23	0.127 ± 0.011

N/A: Not applicable

S2: Material Characterization

Elemental Analysis (CHN): The carbon, hydrogen, and nitrogen content of the different test materials was determined by using a PerkinElmer 2400 Series II CHNS/O Elemental Analyzer (Shelton, CT, USA), and values are presented in Table S2. The amount of filler present in each of the films was confirmed by CHN, considering the theoretical chemical structure of PLA and each of the components.

Table S2. Carbon, hydrogen, and nitrogen content of the tested materials.

Material	ID	% Carbon[a]	% Hydrogen[a]	% Nitrogen[a]
Cellulose powder	Cellulose	42.50 ± 0.34	6.53 ± 0.05	0.04 ± 0.01
Ingeo™ 2003D film	PLA1	50.05 ± 0.05	5.65 ± 0.02	0.01 ± 0.01
	PLA2	49.93 ± 0.11	5.56 ± 0.02	0.01 ± 0.01
	PLA3	49.99 ± 0.05	5.60 ± 0.01	0.01 ± 0.01
Cloisite® 30B	OMMT	19.22 ± 0.06	3.84 ± 0.02	0.99 ± 0.00
Laponite® RD	LRD	0.18 ± 0.01	1.19 ± 0.04	0.02 ± 0.01
Halloysite	HNT	0.09 ± 0.02	1.83 ± 0.05	0.01 ± 0.00
Tomamine™	QAC	59.28 ± 0.60	12.28 ± 0.05	2.55 ± 0.02
PLA-OMMT 1%[a]	PLA-OMMT1	49.49 ± 0.07	5.54 ± 0.04	0.03 ± 0.02
PLA-OMMT 5%[a]	PLA-OMMT5	48.76 ± 0.07	5.49 ± 0.02	0.07 ± 0.01
PLA-OMMT 7.5%[a]	PLA-OMMT7.5	47.75 ± 0.11	5.43 ± 0.01	0.09 ± 0.00
PLA-HNT 1%[a]	PLA-HNT1	49.67 ± 0.12	5.60 ± 0.06	0.70 ± 0.34
PLA-HNT 5%[a]	PLA-HNT5	48.22 ± 0.10	5.44 ± 0.01	1.68 ± 0.43
PLA-LRD 1%[a]	PLA-LRD1	49.58 ± 0.17	5.54 ± 0.05	2.43 ± 0.42
PLA-LRD 5%[a]	PLA-LRD1	47.70 ± 0.11	5.39 ± 0.06	6.43 ± 1.82
PLA-QAC 0.5%	PLA-QAC0.5	49.98 ± 0.08	5.55 ± 0.02	0.01 ± 0.00
PLA-QAC 1.5%	PLA-QAC1.5	50.55 ± 0.04	5.78 ± 0.02	0.05 ± 0.01

[a] Percentage by weight

Differential Scanning Calorimetry (DSC): The glass transition (T_g) and melting (T_m) temperatures of PLA and BNCs films were determined using a DSC model Q-100 (TA Instruments, New Castle, DE) and the TA Instruments Universal Analysis 2000 software (Version 4.5A). The testing temperature was from 5°C to 210°C with a ramping rate of 10°C/min. The results are shown in Figure S1 and Table S3.

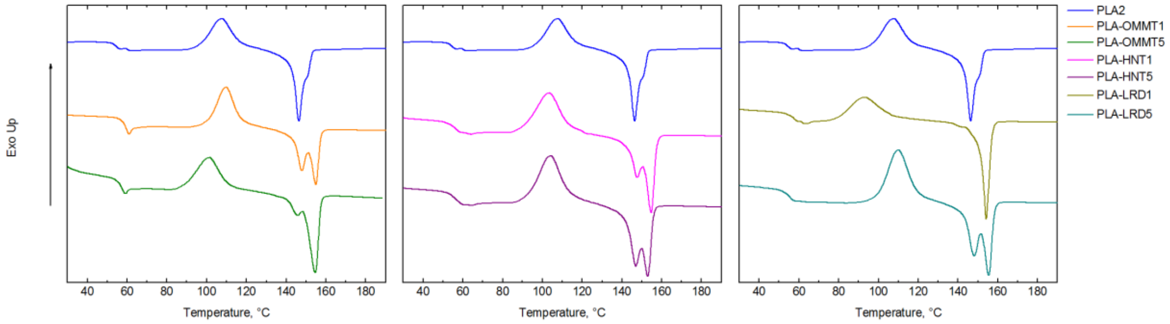

Figure S1. DSC of the PLA and BNCs films (1st cycle).

Thermogravimetric Analysis (TGA): The degradation temperature (T_d) of the PLA and PLA-OMMT films was measured with a TGA model Q50 from Thermal Analysis Inc. (New Castle, DE). The testing temperature was from 23°C to 600°C with a ramping rate of 10°C/min. The results are shown in Figure S2 and Table S3.

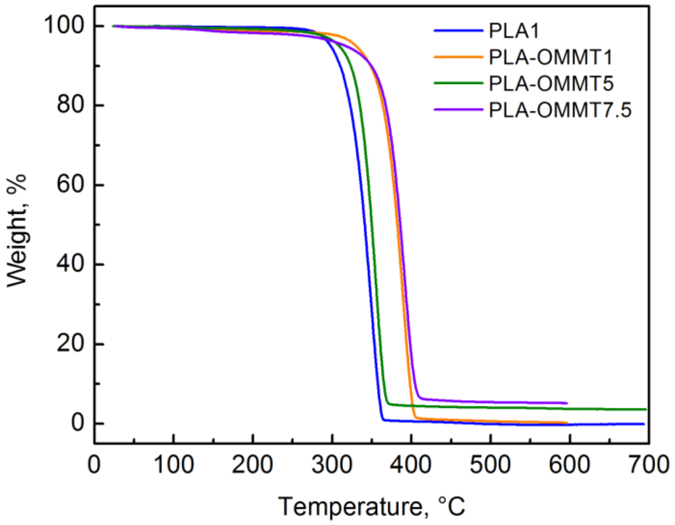

Figure S2. TGA of the PLA and PLA-OMMT films.

Table S3. Thermal properties of the PLA and BNCs.

Sample	T_g, °C	T_c, °C	T_m, °C	T_d, °C	% X_c
PLA1	63.3	N/A	152.0	349.0	25.0
PLA2	54.4	107.1	146.4	N/A	4.6
PLA-MMT1	59.8	109.8	154.9	389.2	1.6
PLA-MMT5	57.8	101.5	154.6	355.0	4.3
PLA-MMT7.5	57.9	90.3	152.8	391.5	12.3
PLA-HNT1	56.8	103.1	154.7	N/A	4.0
PLA-HNT5	56.7	103.7	153.0	N/A	4.5
PLA-LRD1	57.9	92.0	154.2	N/A	11.6
PLA-LRD5	55.6	109.7	155.5	N/A	2.7

N/A: Not available

Moisture sorption isotherm: The moisture sorption isotherms of the nanoclays, PLA, and BNCs films were examined by gravimetric analysis using an SGA-100 from VTI Corp. (Hialeah, FL). The samples (5-10 mg) were exposed to relative humidity (RH) between 0 and 95 ± 2% with RH steps of 10, at 23 ± 0.1°C. The results are shown in Figure S3.

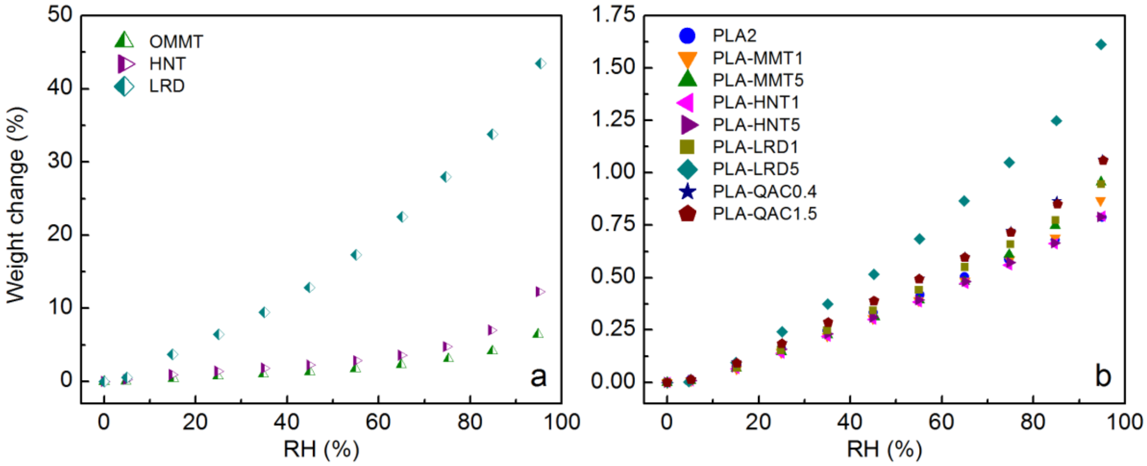

Figure S3. Moisture sorption isotherms of the nanoclays, PLA and BNCs films.

Electrical conductivity: The measurements were carried out using an electrochemical impedance spectroscopy (EIS) system (Gamry Instruments, Warminster, PA) for 2.54 cm^2 film samples. Copper foil tape with conductive adhesive was located on the surface of the film from both sides, and the electrodes were attached to each extreme of the tape. The Gamry Framework software was used for the analysis using the Potentiostatic EIS mode. The conductivity was measured over a frequency range of 1 x 10^5 to 0.1 Hz with an applied potential of 20 mV at room temperature (23°C). The resistivity values presented in Table S4 were calculated using the impedance (Z) value at a frequency of 0.1 Hz.

Table S4. Resisitivity of the PLA and BNCs

Sample	Resistivity			
PLA2	3.96E+13	±	1.47E+11	AB
PLA-OMMT5	3.31E+13	±	5.05E+12	B
PLA-QAC0.4	3.77E+13	±	1.00E+12	AB
PLA-HNT5	3.61E+13	±	4.21E+12	AB
PLA-LRD5	4.15E+13	±	6.20E+11	A

Note: Values with the same letter are not significantly different at $p \leq 0.05$ with Tukey-Kramer Test.

Contact angle: Surface wettability of the PLA and BNCs films was evaluated by contact angle measurements using a goniometer (Drop Shape Analysis System, DSA10 Mk2, Krüss GmbH, Hamburg, Germany), equipped with a diffuse light source and a CCD camera, at room temperature (23°C). A drop of HPLC grade water (3 μL) was deposited on the film surface and a magnified image of the drop profile was conveyed to a computer. The contact angle was measured with the Drop Shape Analysis Software using the tangent method. Ten measurements per film were performed and the values reported in Table S5 are the average of contact angles measured on both sides of the drop.

Table S5. Contact angle of the PLA and BNCs measured with water at room temperature

Sample	Contact angle
PLA2	71.6 ± 2.1 ^{D}
PLA-OMMT5	96.4 ± 4.2 ^{A}
PLA-QAC0.4	83.3 ± 4.5 ^{C}
PLA-HNT5	93.2 ± 2.5 ^{B}
PLA-LRD5	85.6 ± 3.9 ^{C}

Note: Values with the same letter are not significantly different at $p \leq 0.05$ with Tukey-Kramer Test.

S3: Physicochemical characteristics of the compost;

Samples of the compost used in the different biodegradation tests were sent to the Soil and Plant Nutrient Laboratory at Michigan State University (East Lansing, MI, USA) for determination of the physicochemical parameters (dry solids, volatile solids, C/N ratio, pH, and microbial activity) as shown in Table S6. Detailed information about the methods used for compost characterization can be found elsewhere [1].

Table S6. Physicochemical characteristics of the compost used in the different biodegradation tests

Parameters	ISO[b]	I	II	III	IV
Dry solids, %	50-55	53.3	52.7	41.5	60.9
Volatile solids, %	<30	26.4	44.3	43.2	39.1
pH	7-9	7.8	7.9	8.5	7.4
Total Carbon, %	N/A [a]	15.3	25.7	25.1	22.7
Total Nitrogen, %	N/A [a]	0.9	2.4	2.4	2.1
C/N ratio	10-40	17.4	10.8	10.3	10.9
Compost activity [c]	50-150	39.0	81.1	63.0	62.5

[a] Not applicable or not available;

[b] Values based on ISO 14855-1:2005 standard;

[c] Average values measured in mg of CO_2 per g of VS in the first 10 days.

S4: Molecular Weight Determination

Initial molecular weight: The number average molecular weight (M_n), weight average molecular weight (M_w), and polydispersity index (*PDI*) of the samples before and during composting were determined by size exclusion chromatography (SEC) with a system from Waters Inc. (Milford, MA), equipped with a Waters 1515 isocratic pump, a Waters 717 autosampler, a series of three columns (HR2, HR3, and HR4 Waters Styragel®), and a Waters 2414 refractive index detector interfaced with Waters Breeze software [1]. Table S7 shows the initial M_n, M_w, and *PDI* of the samples as measured before each of the different biodegradation tests.

Table S7. Initial M_n, M_w, and *PDI* of the PLA samples.

Biodegradation Test	Sample	M_n, kDa	M_w, kDa	*PDI*
I	PLA1	113.1 ± 0.1 [A]	208.0 ± 0.8 [A]	1.8 ± 0.0 [B]
	PLA-OMMT5	59.8 ± 1.1 [B]	118.9 ± 0.9 [B]	2.0 ± 0.0 [A]
II	PLA1	113.1 ± 0.1 [A]	208.0 ± 0.8 [A]	1.8 ± 0.0 [A]
	PLA-OMMT1	82.9 ± 2.2 [B]	157.3 ± 1.7 [B]	1.9 ± 0.0 [A]
	PLA-OMMT5	59.8 ± 1.1 [C]	118.9 ± 0.9 [C]	2.0 ± 0.0 [A]
	PLA-OMMT7.5	37.5 ± 2.3 [D]	76.7 ± 1.3 [D]	2.1 ± 0.2 [A]
III	PLA2	88.8 ± 0.9 [A]	172.0 ± 1.3 [A]	1.9 ± 0.0 [C]
	PLA-OMMT1	82.9 ± 2.2 [ABC]	157.3 ± 1.7 [B]	1.9 ± 0.0 [C]
	PLA-OMMT5	52.8 ± 0.7 [D]	116.1 ± 0.3 [D]	2.2 ± 0.0 [A]
	PLA-HNT1	91.4 ± 3.3 [A]	171.1 ± 1.2 [A]	1.9 ± 0.1 [C]
	PLA-HNT5	79.7 ± 3.8 [BC]	153.0 ± 2.5 [B]	1.9 ± 0.1 [BC]
	PLA-LRD1	84.2 ± 1.7 [AB]	155.5 ± 1.4 [B]	1.8 ± 0.0 [C]
	PLA-LRD5	75.3 ± 0.9 [C]	139.0 ± 0.7 [C]	1.8 ± 0.0 [C]
	PLA-QAC0.4	43.5 ± 3.8 [E]	88.7 ± 1.6 [F]	2.0 ± 0.1 [ABC]
	PLA-QAC1.5	45.0 ± 2.4 [E]	96.7 ± 1.3 [E]	2.2 ± 0.1 [AB]
IV	PLA1	119.0 ± 11.3 [A]	234.4 ± 16.9 [A]	2.0 ± 0.1 [B]
	PLA2	101.1 ± 11.8 [AB]	206.2 ± 23.1 [A]	2.0 ± 0.1 [AB]
	PLA3	84.8 ± 6.9 [B]	167.4 ± 3.2 [B]	2.0 ± 0.2 [B]
	PLA-OMMT5	45.5 ± 5.8 [C]	108.6 ± 11.6 [C]	2.4 ± 0.2 [A]
	PLA-QAC0.4	54.5 ± 9.5 [C]	118.2 ± 5.8 [C]	2.2 ± 0.3 [AB]

Note: Values with the same letter within the same group (*i.e.*, biodegradation test) and in the same column are not significantly different at $p \leq 0.05$ with Tukey-Kramer Test.

Molecular Weight Reduction during Biodegradation: Due to the observed multimodal MWD in the results presented in Section 3.3 of the text, deconvolution of the MWD peaks was necessary for conducting kinetics analysis, in which the M_n reduction rate (k) constant was calculated for PLA and the BNCs. Therefore, a curve fitting and data analysis program, Fityk version 1.3.0, developed by Marcin Wojdyr [2], was used for deconvolution using a log normal function as was used by Perejon et al., which is more appropriate to fit asymmetrical functions [3] such as the ones observed for the MWD (Figures 13 to 14). Figure S4 shows an example of the deconvolution of the PLA control peaks at day 7, 14, 21, and 28. To confirm whether deconvolution of a peak was necessary or not, and which are the main peaks of the MWD, the area fraction was used. Figure S5 shows the PLA control as an example of the methodology used. Figure S5.a shows the M_n calculated from the different deconvoluted peaks as function of time while Figure S5.b shows the area fraction of those different peaks, in which the first peak has the main contribution until day 21. For PLA control on days 28 and 42, it seems that the first and second peaks may have similar contribution in some cases and the contribution of the other peaks is minimal. In the case of PLA control for day 56 a single peak was observed. This analysis was performed for all the BNCs in a similar fashion and the main peaks were selected case by case for the determination of k. In most cases, no deconvolution was required for days 0, 3, and 7.

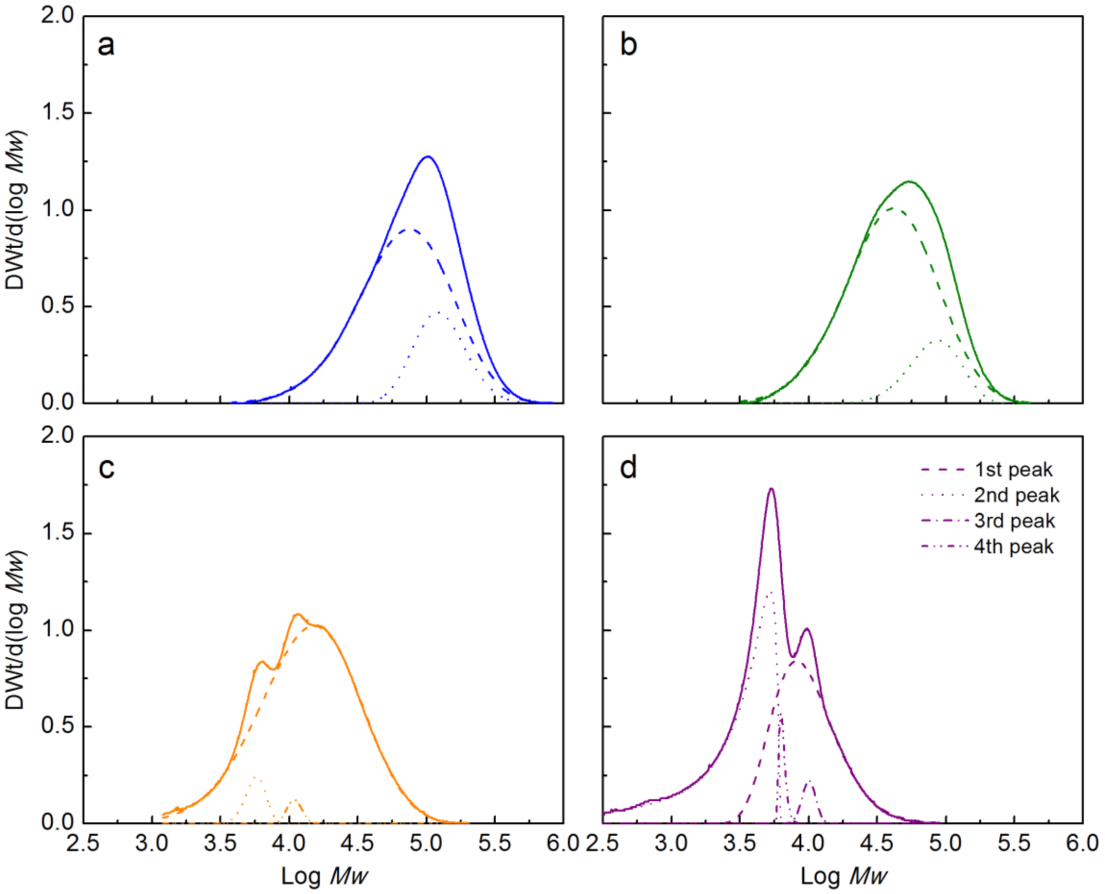

Figure S4. Deconvolution of the PLA2 peaks at days a) 7, b) 14, c) 21, and d) 28 (Test III in compost).

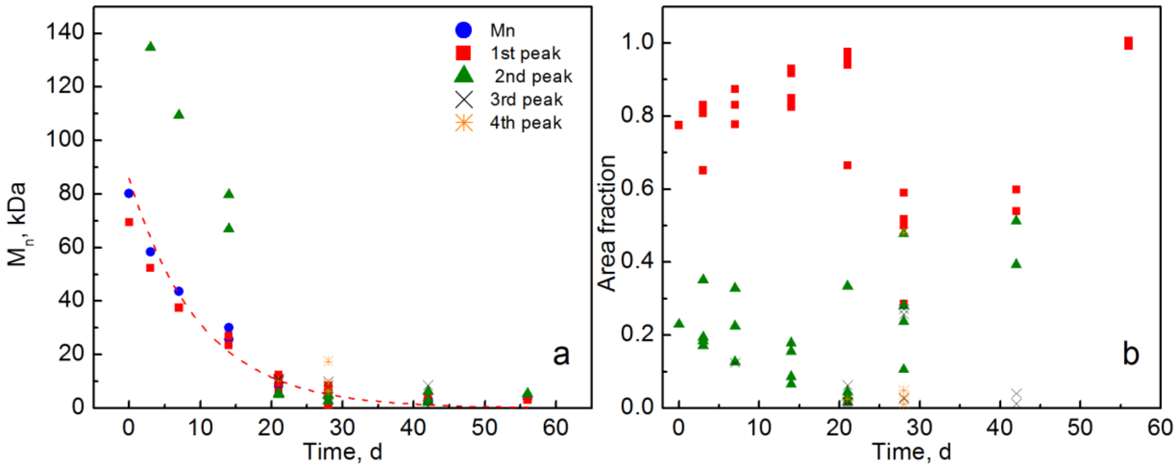

Figure S5. a) M_n and b) area fraction as function of time for PLA2 film (Test III in compost).

Figure S6 and Table S8 show the M_n reduction as a function of time for PLA and PLA-BNCs. The dashed lines indicate fitting of a first order reaction of the form $M_n/M_{n0} = \exp(-kt)$, where M_{n0} is the initial M_n, k is the rate constant and t is the time. It can be observed that the initial molecular weight has a real effect on the biodegradation rate, especially until day 21, in which the abiotic degradation (*i.e.*, hydrolysis) takes place, and therefore the overall biodegradation. A material with low M_n has more polymer chains with free ends that can be cleaved, thus producing more oligomers and monomers that are available for the microorganisms in comparison with one of higher Mn [1]. Figure S6 also shows that for each of the BNCs the film with 1% and 5% filler loading follow a similar pattern. PLA-HNT films (Figure S6.c) are the ones with the closest initial molecular weight to the PLA control and they follow a very similar pattern, especially after the 3rd day. PLA-HNT and PLA-LRD films seem to have a lower rate than the PLA control, which is in agreement with previous results.

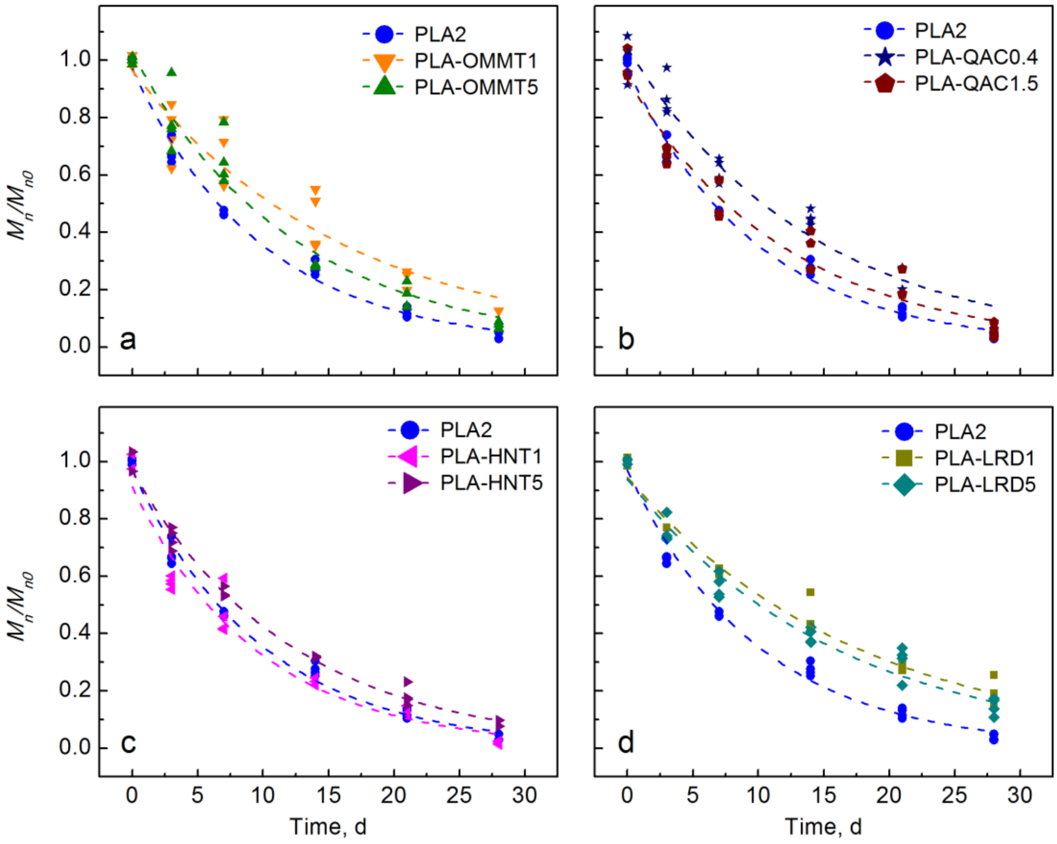

Figure S6. Molecular weight reduction as function of time for PLA2 and a) PLA-OMMT, b) PLA-QAC, c) PLA-HNT, and d) PLA-LRD films (Test III in compost). Dashed lines indicate fitting of a first order reaction of the form M_n/M_{n0}= exp (-*kt*), where M_{n0} is the initial M_n, *k* is the rate constant and *t* is the time.

Table S8. Initial molecular weight and reduction rate of PLA and BNCs as estimated by the first order reaction of the form $M_n = M_{n0}$ exp(-*kt*)

Sample	M_{n0}, kDa	*k*, d^{-1}
PLA2	86.0 ± 1.5 A	0.1008 ± 0.0037 A
PLA-OMMT1	80.0 ± 3.5 ABC	0.0616 ± 0.0058 C
PLA-OMMT5	54.1 ± 1.8 E	0.0815 ± 0.0057 B
PLA-HNT1	83.4 ± 3.3 AB	0.1037 ± 0.0078 A
PLA-HNT5	77.2 ± 1.3 C	0.0824 ± 0.0029 B
PLA-LRD1	79.6 ± 1.8 BC	0.057 ± 0.0027 C
PLA-LRD5	70.7 ± 1.9 D	0.0628 ± 0.0034 C
PLA-QAC04	44.9 ± 1.3 F	0.0711 ± 0.0045 BC
PLA-QAC15	42.8 ± 1.4 F	0.0828 ± 0.0056 B

Note: Values with the same letter within the same column are not significantly different at p≤0.05 with Tukey-Kramer Test.

S5: Biofilm formation;

Figure S7, Tables S9 and S10 show the results of the first iteration of the biofilm test. Looking at the control with PA at 23°C (Figure S7.a), the control wells (R2B – No polymer) had an absorbance (600 nm) of 1.628-2.029 (uninoculated control wells ranged from 0.065 to 0.067). There was no significant difference in the wells supplemented with PLA and BNCs (Table S9). The wells supplemented with PLA-LRD5 had the highest average value of 2.028. At 23°C, *P. aeruginosa* formed

biofilm on the surface of the films. The quantitation of biofilm on PLA ranged from 0.409 to 0.966, which is in accordance with the values observed by Satti et al. [4]. There was no significant difference between PLA and BNCs. However, PLA-HNT5 and PLA-LRD5 showed the highest average values of 1.105 and 1.137, respectively. Then, viewing the total biofilm formed by PA (*i.e.* wells plus films), PLA-LRD5 had the highest average total of 3.165 while the total average for the pristine PLA was 2.390.

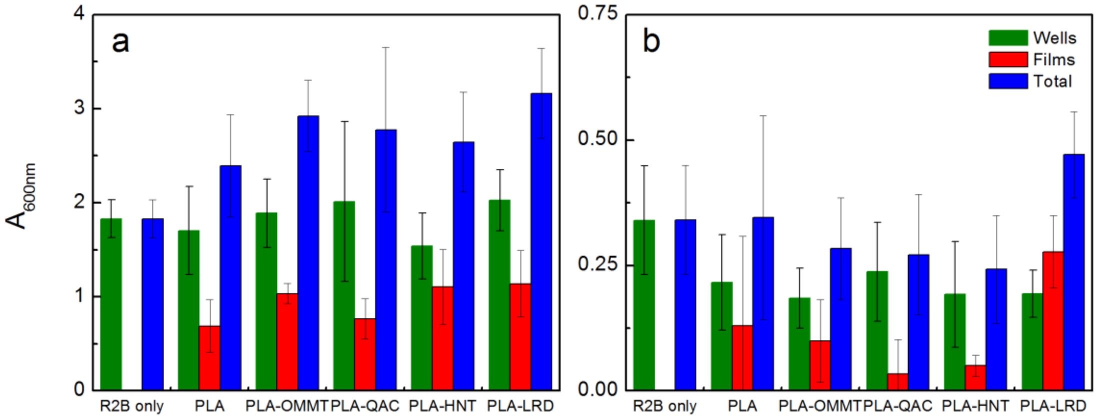

Figure S7. Absorbance (600 nm) of a) PA at 23°C, and b) CE at 58°C first iteration.

Table S9. Absorbance (600 nm) of a) PA at 23°C first iteration

Sample	Wells	Films	Total
w/o PLA	1.829 ± 0.201 A	N/A	1.829 ± 0.201 A
PLA	1.703 ± 0.467 A	0.688 ± 0.279 A	2.390 ± 0.544 A
PLA-OMMT	1.889 ± 0.363 A	1.035 ± 0.108 A	2.924 ± 0.379 A
PLA-QAC	2.012 ± 0.850 A	0.764 ± 0.214 A	2.776 ± 0.876 A
PLA-HNT	1.541 ± 0.351 A	1.105 ± 0.397 A	2.646 ± 0.530 A
PLA-LRD	2.028 ± 0.325 A	1.137 ± 0.353 A	3.165 ± 0.480 A

Note: Values with the same letter within the same column are not significantly different at $p \leq 0.05$ with Tukey-Kramer Test.

Regarding the test with CE at 58°C (Figure S7.b), the sterile controls (SCE) had values that were between 0.54 and 0.57, which were low values considering that the CE still contained humics and other compounds that can bind to polystyrene. The control wells (CE – No polymer) had values of 0.231-0.449. These values were less than the ones for PA at 23°C which was expected since PA is a pure culture of good biofilm former. The wells supplemented with PLA and BNCs look consistent overall in biofilm with values ranging from 0.087-0.312 and no statistically significant difference among them (Table S10). In this case, the control well showed the highest average value of 0.340. PLA-LRD5 has an average value of 0.194. Biofilm formation was detected on PLA and BNCs with CE at 58°C. In this case, PLA-LRD5 has significantly higher value (0.277) than the rest of the BNCs. PLA showed an average value of 0.130 while the lowest average value (0.034) was observed with PLA-QAC0.4. The total biofilm (*i.e.*, wells plus films) was not significantly different among the sample materials.

Table S10. Absorbance (600 nm) of a) CE at 58°C first iteration

Sample	Wells	Films	Total
w/o PLA	0.340 ± 0.109 A	N/A	0.341 ± 0.109 A
PLA	0.216 ± 0.095 A	0.130 ± 0.179 A	0.346 ± 0.203 A
PLA-OMMT	0.185 ± 0.060 A	0.099 ± 0.082 A	0.284 ± 0.102 A
PLA-QAC	0.237 ± 0.098 A	0.034 ± 0.069 A	0.271 ± 0.120 A
PLA-HNT	0.192 ± 0.105 A	0.050 ± 0.021 A	0.242 ± 0.107 A
PLA-LRD	0.194 ± 0.047 A	0.277 ± 0.072 A	0.471 ± 0.086 A

Note: Values with the same letter within the same column are not significantly different at p≤0.05 with Tukey-Kramer Test.

Similarly, Tables S11 and S12 show the results of the biofilm test discussed in Section 3.4 and Figure 16, with PA at 23°C and CE at 58°C.

Table S11. Absorbance (600 nm) of PA at 23°C during the biofilm test

Sample	Wells	Films	Total
R2B only	1.279 ± 0.053 B	N/A	1.279 ± 0.053 CD
PLA2	1.376 ± 0.160 B	0.626 ± 0.125 AB	2.002 ± 0.204 BC
PLA-OMMT5	2.042 ± 0.243 A	0.875 ± 0.089 A	2.917 ± 0.259 A
PLA-QAC0.4	0.977 ± 0.180 B	0.131 ± 0.040 B	1.107 ± 0.185 D
PLA-HNT5	1.044 ± 0.061 B	1.254 ± 0.539 A	2.258 ± 0.542 AB
PLA-LRD5	1.078 ± 0.301 B	0.639 ± 0.097 AB	1.717 ± 0.316 BCD

Note: Values with the same letter within the same column are not significantly different at p≤0.05 with Tukey-Kramer Test.

Table S12. Absorbance (600 nm) of CE at 58°C during the biofilm test.

Sample	Wells	Films	Total
R2B only	0.485 ± 0.103 A	N/A	0.485 ± 0.103 A
PLA2	0.479 ± 0.124 A	0.090 ± 0.030 B	0.569 ± 0.128 A
PLA-OMMT5	0.360 ± 0.238 A	0.175 ± 0.073 B	0.536 ± 0.249 A
PLA-QAC0.4	0.338 ± 0.201 A	0.113 ± 0.032 B	0.451 ± 0.204 A
PLA-HNT5	0.367 ± 0.161 A	0.201 ± 0.014 B	0.568 ± 0.161 A
PLA-LRD5	0.384 ± 0.118 A	0.519 ± 0.054 A	0.903 ± 0.130 A

Note: Values with the same letter within the same column are not significantly different at p≤0.05 with Tukey-Kramer Test.

S6: References.

1. E. Castro-Aguirre, R. Auras, S. Selke, M. Rubino, T. Marsh, Insights on the aerobic biodegradation of polymers by analysis of evolved carbon dioxide in simulated composting conditions. *Polym. Degrad. Stab.* 2017, 137, 251–271. doi:10.1016/j.polymdegradstab.2017.01.017.
2. M. Wojdyr, Fityk: A general-purpose peak fitting program. *J. Appl. Crystallogr.* 2010, 43, 1126–1128. doi:10.1107/S0021889810030499.
3. A. Perejón, P.E. Sánchez-Jiménez, J.M. Criado, L.A. Pérez-Maqueda, Kinetic Analysis of Complex Solid-State Reactions. A New Deconvolution Procedure. *J. Phys. Chem. B.* 2011, 115, 1780–1791. doi:10.1021/jp110895z.
4. S.M. Satti, A.A. Shah, R. Auras, T.L. Marsh, Isolation and characterization of bacteria capable of degrading poly(lactic acid) at ambient temperature. *Polym. Degrad. Stab.* 2017, 144, 392–400. doi:10.1016/j.polymdegradstab.2017.08.023.
